# 

**Published:** 1841-07

**Authors:** 


### CONTENTS

OF NO. I.

### ORIGINAL COMMUNICATIONS.

### ESSAYS AND CASES.

ART. I.—Remarks on Plethora, read to the Medical Society of Tennessee, at its annual meeting, in May, 1841. By SAMUEL HOGG, M. D., President of the Society. 1

ART. II.—Diaphragmatic Hernia. By GEORGE W. BAYLESS, M. D., Demonstrator of Anatomy in the Medical Institute of Louisville. - - - - - - - 14

ART. III.—A Case of Gun Shot Wound involving the Colon, Small Intestines and Right Kidney, attended by recovery. Reported to the Medical Society of Tennessee, at its annual meeting in May, 1841. By JOHN W. RICHARDSON, M. D., of Rutherford County, Tennessee. - - - 28

ART. IV.—Cases of Wounded Arteries of the Arm, relieved by the Bandage. Reported to the Medical Society of Tennessee, May, 1841. By DR. GEORGE THOMPSON, of Jefferson, Tennessee. - - - - - - - 33

ART. V.—Notes of a Singular Case of Fatal Abdominal Disease. By Dr. J. J. POLK, of Perryville, Ky. 34

### REVIEWS.

ART. I.—Brief Sketch of the Life and Services of John Hunter. Condensed from his Biography by Drewry Ottley. London. 37
Bibliographical Notices. - - - - - 51–52

### SELECTIONS FROM AMERICAN AND FOREIGN JOURNALS.

Observations on Ergot. By JOHN B. BECK, M. D., Professor of Materia Medica and Medical Jurisprudence in the College of Physicians and Surgeons of New York. - - - - 53
History of the last Illness of Sir A. P. Cooper, Bart., and examination of the body after death. - - - - - 65
On Effusions into the Cavity of the Thorax, and a new method of evacuating them. By M. REYBARD. - - - - 72

### ORIGINAL INTELLIGENCE.

Contributions to the Pathological Museum of the Medical Institute of Louisville. - - - - - - - 73
Contributions to our Journal by Societies. - - - - 75
Milk Sickness. - - - - - - - 75
New Cure for Hydrocele. - - - - - - 76
Fever. By M. L. LINTON, M. D. - - - - - 77
Tenotomy. - - - - - - - 80
Transylvania University. - - - - - - 80
Obituary. - - - - - - - 80

